# Efficacy of Laser Therapy for Grade C Periodontitis in Young Individuals

**DOI:** 10.7150/ijms.107543

**Published:** 2025-05-28

**Authors:** Sijia Liu, Wanqing Zhao, Yusen Qie, Na Liu, Qing Liu

**Affiliations:** 1 Hebei Key Laboratory of Stomatology/ Hebei Technology Innovation Center of Oral Health, School and Hospital of Stomatology, Hebei Medical University, Shijiazhuang, China; 2 Department of Preventive Dentistry, School and Hospital of Stomatology, Hebei Medical University, Shijiazhuang, China

**Keywords:** aggressive periodontitis, laser, antibacterial photodynamic therapy (APDT), therapy

## Abstract

Grade C periodontitis in young individuals is characterized by its early onset and rapid progression, resulting in swift periodontal tissue destruction in systemically healthy individuals. The application of laser technology in periodontal therapy has gradually increased in recent years. Laser therapy offers several advantages over traditional antibiotics, such as reduced patient discomfort, minimized postoperative edema, and a lower risk of drug-resistant strains. Recent studies suggest that laser-assisted treatments can significantly augment the clinical efficacy in managing grade C periodontitis. However, available evidence has not drawn distinct conclusions. This review aims to present the research progress in laser and antibacterial photodynamic therapy as the adjuvant treatment of grade C periodontitis in young individuals over the past decade, offering references for clinical practice.

## 1. Introduction

Grade C (high-risk) periodontitis, as described by Papapanou et al. (2018) 1, is commonly referred to as aggressive periodontitis according to Armitage (1999) 2. It features the rapid destruction of periodontal tissues caused by disproportionate local irritation, a swift deepening of periodontal pockets, and accelerated resorption of alveolar bone. The disease may be localized when primarily impacting the first molars and incisors, now referred to as grade C molar-incisor pattern periodontitis, previously termed localized aggressive periodontitis (Armitage, 1999). However, when the disease extends to involve additional teeth, it is termed generalized grade C periodontitis 3, formerly termed generalized aggressive periodontitis (Armitage, 1999) (Table 1) 4. Grade C periodontitis is the updated term for aggressive periodontitis. Although the term aggressive periodontitis is considered outdated, it is still referenced in the historical background in this review.

Currently, non-surgical treatment approaches, including subgingival scaling and root planning (SRP), remain the primary treatment options. Nevertheless, the intricate anatomy of teeth and limitations of therapeutic instruments pose challenges in completely removing calculus and plaque in areas like deep periodontal pockets and root furcation. Surgical periodontitis treatment (SPT) has demonstrated advantages in addressing these limitations by offering improved visibility and facilitating device operation. Nonetheless, residual diseased tissue and bacteria in the periodontal soft tissue wall may still not be entirely eradicated, which tends to affect the attachment of gingival connective tissue to the root surface, consequently hindering periodontal tissue regeneration. The systemic administration of antibiotics such as amoxicillin and metronidazole has shown effectiveness in eliminating tissue-invasive bacteria such as *Aggregatibacter actinomycetemcomitans (A.a)* and *Porphyromonas gingivals (P.g)*, but their efficacy is limited and susceptible to drug resistance 5. In previous studies, the resistance of *P.g* to amoxicillin, the primary periodontal pathogen affecting American patients with periodontitis, increased from 0.1% in 1999-2000 to 2.8% in 2019-2020 6. Additionally, metronidazole resistance, which was once considered rare, has steadily risen over the past 20 years 7. Recent studies have also revealed that, in addition to the resistance of *A.a* to amoxicillin, other periodontal pathogens have exhibited resistance to tetracycline and tinidazole 5,8. Researchers have been exploring novel adjunctive or alternative therapies. Advancements of dental laser technology have introduced new possibilities and perspectives for treatment of periodontitis, offering innovative approaches to address these challenges. Laser therapy can effectively eliminate bacteria in periodontal pockets, reduce the levels of inflammatory factors, and promote tissue repair and regeneration, all while circumventing the limitations of antibiotic therapy 9-13 (Table 2). Consequently, laser-assisted therapy is anticipated to serve as a valuable complement to traditional treatment methods, offering more precise and personalized solutions for the management of periodontitis.

The review aims to show and discuss studies regarding the efficacy of laser and antimicrobial photodynamic therapy in treating young individuals with grade C periodontitis. The changes in the classification and related terminology of grade C periodontitis, as well as the limited number of studies on different disease types, render the results ambiguous and incomparable. However, the existence of these research gaps primarily arises from several factors: (1) the complexity and variety of disease types; (2) the diversity of laser technical parameters, such as laser wavelength and energy density; (3) a lack of long-term efficacy and safety evaluation data; and (4) the high cost of laser equipment, coupled with limited research resources, which may weaken studies on laser treatment for rare types of periodontitis. These challenges hinder the comprehensive development of the field and restrict standardized application and clinical technology promotion. Furthermore, they delay the update of clinical guidelines and technological innovations related to laser therapy. Therefore, to further distinguish three phenotypes of periodontitis 14and then provide targeted treatment measures, the review focuses on recent studies from the last decade that evaluate the efficacy of laser-assisted therapy in individuals younger than 35 years, diagnosed with grade C periodontitis and without other risk factors such as diabetes and smoking (Table 3). In this review, authors strictly limit the inclusion criteria for aggressive periodontitis cases in the study, while young individuals who were previously diagnosed with aggressive periodontitis are now diagnosed with grade C periodontitis, and this new term will be used.

## 2. Overview of the laser

Since the advent of laser technology, it has rapidly advanced in the medical field and has increasingly become a focus of new research. In 1964, Goldman L et al. first mentioned the application of laser technology in dentistry 15, marking the inception of the application of lasers in stomatology. Since then, different types of lasers have been developed and applied in stomatology. With its unique advantages, including easy operation, decreased patient injury, efficacious hemostatic effect, and reduced pain response, laser technology has progressively evolved into an adjunctive and alternative therapy for periodontal fundamental therapy. The treatment and prognosis of periodontitis are relatively complex, with studies on the application of laser in its treatment having been documented since 1988 16. In recent years, the commonly used laser types are Diode laser (DL), Pulsed Neodymium-doped Yttrium Aluminum Garnet (Nd: YAG) laser, dual-wavelength laser, Erbium-doped Yttrium Aluminum Garnet (Er: YAG) laser, and Erbium, Chromium-doped Yttrium Scandium Gallium Garnet (Er. Cr: YSGG) laser. The study has shown that integrating laser with SRP in periodontitis treatment leads to significant short-term therapeutic efficacy 17.

## 3. Applications of various lasers

### 3.1 Diode Laser (DL)

The wavelength of diode laser (DL) typically ranges from 800 to 990nm, approaching the absorption peaks of hemoglobin and melanin, which qualifies it as a low-energy laser. Moreover, it exhibits a high absorption rate for water and hydroxyapatite 11. Its working principle is based on photothermal, biological stimulation, and mechanical effects to achieve sterilization and bacteriostasis, facilitate the resolution of inflammation at the lesion site, enhance the local microcirculation, and provide hemostatic and analgesic effects 18.

Many studies have shown that DL, as an adjunct SRP, can improve the relevant periodontal clinical indices in the short term and further enhance the efficacy of fundamental periodontal therapy 19-22. Meanwhile, DL treatment for grade C periodontitis in young individuals exhibits an outstanding antibacterial effect. In 2022, Doğan ^B et.al discovered that based on SRP, DL laser-assisted modified Widman flap (MWF) can effectively reduce bacteria in the periodontal pocket. It can also overcome the limitations of traditional surgery treatment, such as the difficulty in removing epithelial remnants and tissue-invasive pathogens. Consequently, this confirms that the use of DL as a supplement to SRP is feasible during surgical treatment, which helps improve the effect of surgical treatment 18. In 2024, Anwar SK et al. compared DL laser treatment with systemic antibiotics, and the findings revealed that* P. g* and *A.a* levels steadily decreased after 3-month in the test group, while the control group slightly increased, suggesting that DL may avoid the issue of antibiotic resistance 23. Conversely, Matarese G et al. in 2017 reported that while SRP combined with 810 nm DL laser treatment notably reduced some periodontal clinical parameters 1-year post-treatment compared to SRP alone, the decrease of microorganisms and inflammatory mediators was not statistically significant 24. It could be attributed to the absence of oral hygiene measures and the re-colonization of microorganisms over an extended period, further impairing the long-term efficacy of DL as an adjuvant therapy. Thereby, this highlights the necessity of periodontal maintenance therapy for patients with periodontitis.

Numerous variable factors in clinical procedures, including the duration of laser exposure, energy density, projection angle, and wavelength, influence the therapeutic outcomes. It is noteworthy that the wavelengths of DL lasers used in current studies are not uniform, and the optimal wavelength for the most effective therapeutic outcome for young individuals with grade C periodontitis is yet unclear. Although DL has demonstrated certain advantages over antibiotic treatment, its safety and potential to replace antibiotic therapy remain undetermined. Additionally, further research with extended follow-up periods is essential to verify the long-term efficacy of DL.

### 3.2 Neodymium-doped Yttrium Aluminum Garnet (Nd: YAG) Laser

Nd: YAG, a neodymium laser with a wavelength of 1064 nm, is optimally absorbed by soft tissue. Its activation medium is neodymium-doped yttrium aluminum garnet. It is absorbed by melanin and hemoglobin 25, exhibiting a strong bactericidal effect and providing efficacious hemostasis and analgesia. Additionally, it can cut soft tissue, cleanse the epithelial lining of periodontal pockets, and vaporize the pockets without causing damage or carbonization of connective tissue 26, making it extensively utilized in soft tissue treatment. However, high-energy lasers have the potential to damage periodontal hard tissue, resulting in rough, depressed root surfaces and carbonization 27. Consequently, it is crucial to carefully manage laser parameters during operation to prevent thermal side effects. Research conducted by Tan Yani et.al in 2022 has demonstrated 28 that Nd: YAG laser-assisted flap surgery offered remarkable therapeutic benefits for patients with generalized grade C periodontitis. It could significantly decrease periodontal indices and pathogenic bacteria levels while increasing the levels of nuclear factor-kB receptor activator ligand (RANKL) / osteoprotegerin (OPG) in the gingival crevicular fluid. These outcomes exceeded those of traditional flap surgery.

Currently, there are limited studies on the use of Nd: YAG laser as adjuvant therapy for grade C periodontitis in young individuals. Furthermore, there is a lack of comparative studies evaluating the merits and drawbacks of Nd: YAG laser versus SRP. The efficacy of Nd: YAG adjuvant therapy and whether it can replace the treatment remains to be further investigated.

### 3.3 Er: YAG Laser and Er. Cr: YSGG Laser

#### 3.3.1 Er: YAG Laser

Er: YAG, an erbium laser activated by erbium yttrium aluminum garnet, operates at a wavelength of 2940nm, close to water's absorption peak at 2950nm. It is readily absorbed by water and hydroxyapatite in the dental hard tissues. The Er: YAG laser can remove dental calculus, smear layers, and diseased cementum without causing damage to the dentin, resulting in minimal to no thermal side effects on the root surface 27,29. Concurrently, it eliminates bacteria from the cementum surface, alleviating gingival inflammation and promoting periodontal healing 30. Compared to the Nd: YAG laser, the primary advantage of the Er: YAG laser is the minimal thermal side effects when removing diseased hard tissues, thereby considerably mitigating damage to adjacent tissues 31. In a study conducted by Shi Xuexue in 2021 32, significant improvements in periodontal clinical indicators such as Plaque Index (PI), Bleeding on Probing (BOP), and clinical attachment level (CAL) were observed in the short-term in the group treated with adjunctive laser therapy, compared to the SRP group.

The study validated the efficacy of Er: YAG laser treatment for grade C periodontitis. However, given the limited follow-up period, more multi-center trials with larger sample sizes are required for a more comprehensive assessment of long-term outcomes. Subsequent research may concentrate on the influence of Er: YAG laser on high-risk pathogenic bacteria and the host response in grade C periodontitis, aiming to provide further insights for clinical practice.

#### 3.3.2 Er. Cr: YSGG Laser

Similar to the Er: YAG, Er. Cr: YSGG is an erbium laser activated by erbium-chromium yttrium scandium gallium garnet, with a wavelength of 2780 nm. The Er. Cr: YSGG laser can be applied for soft and hard tissue applications and function on hydrodynamic principles to alleviate inflammation and eliminate periodontal pathogens 33,34. It also removes the smear layer, safeguards the deep healthy cementum, and prevents root sensitivity, pulp inflammation, and tissue damage due to dentine exposure 29. Compared to the Er: YAG laser, the Er. Cr: YSGG laser exhibits a higher absorption rate in water and hydroxyapatite, enabling more efficacious removal of dental calculus. Moreover, its optimal tissue penetration rate effectively seals capillaries, providing a hemostatic and analgesic effect 29,35,36.

Several studies have confirmed the therapeutic efficacy of Er. Cr: YSGG laser in generalized grade C periodontitis 19-21,37. Laser-assisted SRP treatment can effectively improve key periodontal clinical parameters, including a reduction in pocket depth (PD), clinical attachment loss (CAL), and bleeding on probing (BOP), and lowered the levels of inflammatory factors in the gingival crevicular fluid. The findings suggested that Er. Cr: YSGG laser might be more appropriate for treating generalized grade C periodontitis than DL laser. In 2022, Talmac AC et al. 19 found that the combination of Er. Cr: YSGG laser and SRP in treating generalized grade C periodontitis was superior to other modalities. This approach resulted in a reduction of probing depth (PD), clinical attachment level (CAL), bleeding on probing (BOP), and plaque index (BI) values, as well as more significant decreases in levels of Tumor Necrosis Factor-alpha (TNF-α), Interleukin-1β (IL-1β), and Interleukin-8 (IL-8). Corresponding studies further support this conclusion 20,21. In contrast to DL treatment and SRP alone, Er. Cr: YSGG laser treatment decreased the gingival crevicular fluid and significantly reduced the inflammatory cytokines.

Future studies should concentrate on the effect of Er. Cr: YSGG laser on high-risk periodontal pathogens associated with grade C periodontitis and prolong the follow-up period to evaluate the long-term outcomes. Most studies employed a split-mouth design, which inevitably entailed a carry-over effect among each other and potentially influenced the research outcomes. Therefore, it is advisable to enlarge the sample size, consider modifying the experimental design, and adopt randomized controlled trials to enhance the credibility of the study outcomes.

### 3.4 Dual- wavelength Laser (Er: YAG Laser and Nd: YAG Laser)

Studies have shown that single laser-assisted SRP is effective in treating young individuals with grade C periodontitis. However, there remains an issue that fails to address the soft and hard tissues within the periodontal pocket. Integrating with diverse wavelengths and characteristics of the lasers enables leveraging their respective advantages to maximize benefits and minimize drawbacks, thereby leading to an optimal therapeutic outcome. Research has demonstrated that dual-wavelength lasers offer synergistic effects, significantly improving patients' clinical symptoms, controlling periodontal inflammation, and facilitating periodontal tissue regeneration 26. Recent studies have explored the effectiveness of Er: YAG and Nd: YAG lasers as an adjunctive treatment for grade C periodontitis. In 2019, the research by Zheng Ying et al. on 12 patients with generalized grade C periodontitis 38 and another study by Yang Ting et al. involving 78 patients 39 indicated that the dual-laser group could further enhance periodontal clinical parameters in the short term. The results surpassed those of both the single-laser and SRP groups. Nevertheless, the long-term efficacy requires further study. Additionally, investigating the combined application of different types of lasers can provide more clinical treatment alternatives for patients.

### 3.5 Antimicrobial photodynamic therapy (APDT)

The treatment of periodontitis using APDT relies on three key components: the light source, photosensitizer, and molecular oxygen within the tissue, working synergistically. The principle of this approach entails photoactive substances (photosensitizers) binding to target cells, followed by activation with specific wavelengths of light, leading to the generation of singlet oxygen free radicals with subsequent cytotoxic effects on the cells 40,41. Methylamine blue, methylene blue, indocyanine green, and curcumin are commonly used photosensitizers in APDT 41,42. The advantages of APDT encompass its simplicity, minimal patient trauma, repeatability, and non-development of resistance 43. APDT can rapidly eliminate microbial cells, and it is particularly effective against antibiotic-resistant biofilm infections, such as* P. g* and *Enterococcus faecalis (E.f)*, in contrast to antibiotics and antifungals that may take several days to exhibit efficacy. Nowadays, there is controversy regarding the studies of APDT in treating young individuals with grade C periodontitis.

Novaes AB et al. found that APDT and SRP affected different species of bacteria in treating grade C periodontitis. APDT demonstrated superior efficacy against *A.a*, while SRP was more effective against red complex pathogens such as *Tannerella forsythia (T.f)* and *P. g* 44. Thus, it is reasonable to hypothesize that APDT combined with SRP may be beneficial in treating grade C periodontitis. In 2022, Chawla K et.al has showed that APDT-assisted SRP treatment for grade C periodontitis has an advantage in increasing CAL compared to SRP alone at 3 and 6 months 37. In 2023, Rodrigues RD et al. also found that APDT-assisted SRP was significantly more effective than SRP in treating grade C periodontitis after 3 months, particularly in sites with a baseline probing depth (PD) ≥ 4 mm 45. However, the results of the studies are inconsistent. A randomized controlled study by Borekci T et al. in 2019 explored the use of APDT as an adjunct to non-surgical periodontal therapy in young individuals with grade C periodontitis. In addition to sulcus bleeding index (SBI), APDT was not superior to SRP in terms of periodontal clinical and microbiological parameters investigated after 63 days, but it confirmed that APDT may provide additional benefits in reducing gingival bleeding 46.

The research by Andere NMRB et al. in 2022 compared the efficacy of repeated APDT as an adjunctive treatment for grade C periodontitis to open flap debridement (OFD) 47. Researchers found that both could significantly improve periodontal clinical parameters and decrease the number of *P. g*; however, each approach presented unique advantages. Although both approaches demonstrated a statistically significant reduction in moderate-depth periodontal pockets, the OFD group with the deep periodontal pockets exhibited a more considerable reduction in PD and a rise in CAL at all evaluated time points. After 1-year treatment, Gingival recession (GR) in the APDT group was significantly less than that of the OFD group, exhibiting a superior aesthetic effect and reducing patient discomfort and dentin sensitivity. Furthermore, it suggested that employing APDT rather than OFD within 14 days after periodontal debridement could improve host accommodations. Patient opinions and satisfaction play a significant role in selecting treatment options. Considering grade C periodontitis patients are young and require high aesthetic standards post-treatment, APDT appears to be an effective alternative for the treatment of moderate periodontal pockets in young periodontitis patients. However, other minimally invasive surgical treatment options require further comparisons to evaluate the relative efficacy and applicability of APDT.

Some scholars have compared the efficacy of APDT and antibiotics combined with SRP in treating grade C periodontitis and explored the potential benefits of their combined use. In 2018, Bechara AN et al. showed 48 that all treatment regimens achieved excellent efficacy, with decreased PD, BOP, and increased CAL at the 6-month. The ultrasonic periodontal debridement (UPD) combined with clindamycin (CLM) group showed a more notable reduction in mean probing depth than the APDT group, and the percentage of residual periodontal pockets was lower than the APDT group, suggesting a superior therapeutic effect of antibiotics over APDT. Additionally, there is no additional benefit from combining APDT with antibiotics (CLM). Another study 49 conducted by Al-Khureif AA in 2020 showed that antibiotics (metronidazole and amoxicillin, MTZ + AMX) may more effectively lower pro-inflammatory cytokines than APDT with SRP. It cannot deny the benefit of APDT combined with SRP in treating grade C periodontitis. However, limited evidence has demonstrated that APDT may be less effective than antibiotics. It is important to note that no study has shown that using APDT, which has few side effects and high reproducibility, causes resistance similar to that of antibiotics. Nevertheless, the clinical feasibility of APDT as an alternative to antibiotics still necessitates a thorough evaluation. In the later stages, future research can concentrate on the impacts of both therapies on periodontal pathogens to comprehensively assess their benefits and drawbacks.

In 2018, Andere NMRB et al. also found a significant reduction in PD after treatment in the UPD combined with APDT group. However, the results were not superior to the UPD group 48, showing that a single application of APDT may be insufficient to achieve additional improvements in PD reduction and attachment level (AL) gain. Consequently, the authors believe that the efficacy of APDT-assisted SRP in treating grade C periodontitis may be related to the frequency of applications. A short-term randomized controlled study by Annaji S in 2016 demonstrated that APDT when administered alone, outperformed simple laser irradiation in the adjunctive treatment of grade C periodontitis. Additionally, multiple applications of APDT could produce additional benefits compared to a single application 22. As reported by Costa Coelho TDR et al. in 2023, after 90 days, a significant improvement in BOP and PD was observed in the SRP combined with twice applications of the APDT group for grade C periodontitis in molars compared to SRP alone 50. Furthermore, in 2015, Moreira AL et al. found that the combination of SRP and repeated APDT (four times) further decreased PD, reduced the number of red and orange complexes, and increased clinical attachment of deep periodontal pockets within 90 days compared to SRP alone 51. A single APDT is insufficient to eradicate all periodontal pathogens, which allows bacteria to recolonize and proliferate, thus affecting the efficacy of APDT-assisted SRP in treating grade C periodontitis. Although it remains unclear whether repeated applications of APDT lead to negative consequences, the hypothesis is supported within the limited study scope. Given the relatively short follow-up time of the study, the longitudinal observation period in the future could be extended to ascertain whether APDT combined with SRP would produce sustained beneficial changes over time in the analyzed parameters examined.

The efficacy of APDT adjuvant therapy may be related to periodontal pocket depth. Studies demonstrated that APDT exhibited better efficacy in reducing deep periodontal pockets with PD ≥ 7 mm than moderate-depth periodontal pockets 49,51. The reason may be that mechanical treatment methods, such as SRP, make it difficult to remove bacteria and calculus from deep areas. However, APDT employed soft laser irradiation combined with photosensitizers, overcoming these limitations and enhancing therapeutic outcomes.

The included investigations vary in the laser wavelength, irradiation duration, fiber diameters, and photosensitizers used. Diode laser is the most frequently used light source, and toluidine blue and methylene blue are commonly used photosensitizers. Whether light sources and photosensitizers are superior or inferior remains to be clarified. Furthermore, the optimal parameters, including the irradiation duration, frequency of application, and time intervals for APDT irradiation, require further research to establish the best treatment strategy.

### 3.6 Laser-Assisted New Attachment Procedure (LANAP)

Laser-Assisted New Attachment Procedure (LANAP) is a minimally invasive, closed-pocket surgical therapy introduced by Gregg and McCarthy in 1990 and approved by the U.S. Food and Drug Administration (FDA) in 2004 52. LANAP is indicated for patients with periodontal pocket depths of 4 mm or greater and is recognized as an effective laser-assisted surgical method for treating moderate to advanced periodontitis 53. One of the most significant advantages of LANAP is its capacity to achieve true periodontal tissue regeneration. LANAP induces pocket closure by generating thermally denatured fibrin clots that serve as a physical barrier, preventing epithelial down-growth toward the root apex. Additionally, it promotes healing in an apical-to-coronal direction by stimulating the release of multipotent cells from the alveolar bone and periodontal ligament 54.

Bechir ES compared the clinical outcomes of LANAP with those of scaling and root planing (SRP). The researcher found that LANAP resulted in greater reductions in PD, BOP, and plaque levels, yielding overall superior outcomes compared to SRP alone 55. Although LANAP may exhibit delayed vascular reorganization during the initial stages of wound healing, it demonstrates significant benefits in reducing subgingival periodontal pathogens 56 and minimizing patient bleeding, edema, and postoperative discomfort 57. The ultimate goal of periodontal therapy is tooth retention. The study has shown that full-mouth LANAP treatment achieves comparable tooth retention rates to traditional periodontal surgery or non-surgical therapy 58.

Despite these promising results, no clinical reports have yet documented the application of LANAP in treating young patients with Grade C periodontitis. Given its advantages in minimally invasive procedures, reduced bleeding, and enhanced periodontal tissue regeneration, LANAP holds potential as an adjunctive tool to conventional periodontal therapy for young patients with Grade C periodontitis. Future research should focus on evaluating the efficacy of LANAP in Grade C periodontitis, particularly regarding its long-term outcomes, tissue regeneration capacity, and ability to control disease progression in young patients. However, there is a lack of large-scale, randomized controlled trials comparing it with other minimally invasive therapies, such as photodynamic therapy and ultrasonic-guided subgingival debridement. Additionally, further optimization of LANAP laser parameters and treatment protocols is necessary, along with exploring combination therapies, such as those involving antibiotics. It can enhance treatment efficacy, reduce patient discomfort, and provide more promising therapeutic options for young patients with Grade C periodontitis.

## 4. Conclusions

In summary, there exist few studies on laser treatment for grade C periodontitis in young individuals, both domestically and internationally. Various scholars have produced differing results and perspectives regarding this issue. The discrepancies potentially stem from variations in laser parameters, frequency of laser applications, changes in grade C periodontitis classification, and associated terms. Based on the limited research, it can be tentatively inferred that laser-assisted treatment for young patients with grade C periodontitis is effective and has a promising future in clinical applications.

Some research limitations, including the inclusion of few participants due to the low incidence rate, split-mouth design, and single-center trials, caused bias in the research findings. Previous studies have confirmed that laser therapy demonstrates significant long-term efficacy in treating periodontitis, effectively improving clinical indicators and remaining stable for up to 24 months after treatment 59. However, the observation period in the included studies is predominantly 12 to 24 weeks, which is relatively short. Consequently, there is still a lack of related evidence regarding the long-term efficacy of laser treatment for young patients with grade C periodontitis. To further validate the study results, it is advisable to expand the sample size, consider a prospective, randomized, single-blind, multicenter trial, and prolong the longitudinal observation period to evaluate the long-term efficacy. To date, no study has pointed out the most effective laser or identified a set of optimal laser parameter settings, including laser wavelength, power, output energy, pulse frequency, fiber diameter, and irradiation time. Further research is required to standardize these laser parameters to establish more systematic treatment protocols. Future research should aim to standardize the laser parameters further, extend the follow-up period, and investigate combined laser therapy strategies to provide more systematic treatment options for young patients with grade C periodontitis.

## Figures and Tables

**Table 1:** Terminology Update

	Grade C Periodontitis (2018)	Aggressive Periodontitis (1999)
**Classification System**	The staging and grading system in the 2018 classification of periodontal disease, emphasizes the risk and prognosis of disease progression, with Grade C indicating a high risk of progression.	An independent classification in the 1999 International Classification of Periodontal Diseases (ICPD) emphasizes the rapid progression and early onset of the disease.
**Terminology Definitions**	Classified into three levels: A (slow progression), B (moderate progression), and C (rapid progression), according to the medical history analysis, the progression rate, assessment of risk factors, prognosis evaluation, and impact on the patient's overall health. Grade C corresponds to aggressive features.	It includes the original terms "adolescent periodontitis," "rapidly progressive periodontitis," and other types.
**classification**	Grade C molar-incisor pattern periodontitis; localized Grade C periodontitis; generalized Grade C periodontitis.	Localized aggressive periodontitis; generalized aggressive periodontitis.
**Age**	Provide a more detailed description based on the specific stage, either III or IV.	These conditions occur in teenagers and young adults under the age of 35.

**Table 2:** Laser therapy vs. Antibiotic Therapy

	Laser therapy	Antibiotic Therapy
**Mechanism of Action**	Utilizing a photosensitizer or a specific wavelength of light to disrupt bacterial structures through mechanical, biological, and photothermal effects.	Inhibit bacterial cell wall synthesis, interfere with bacterial protein synthesis, or destroy bacterial DNA, among other mechanisms.
**Targeting**	Targeted Pathogens	Relying on antimicrobial spectrums may disrupt the normal flora.
**Risk of Drug Resistance**	There is a low likelihood of medication resistance because the mode of action is not related to bacteria's typical metabolic pathways.	High Risk of Drug Resistance
**Security**	The precise impact on the affected area occurs without any noticeable side effects.	Systemic side effects may occur, including allergic reactions and potential damage to liver and kidney function.
**Onset Time**	Quick Onset	Slow Onset of Systemic Application
**Treatment Process**	Local application, typically used in conjunction with periodontal scaling.	Typically, it should be administered locally or orally, adhere to the prescribed course of medication, and rely on patient compliance.
**Recovery Period**	Shorten recovery time, relieve pain, and enhance wound healing.	The recovery time is prolonged, and the effectiveness is contingent upon the sensitivity of the antibiotics.
**Joint Application Potential**	The combination of certain antibiotics can enhance the antibacterial effectiveness.	Drug interactions must be taken into account when implementing combination therapy.

**Table 3:** Summary of Studies Showing Clinical or microbiological parameters and Host Response for Localized and Generalized Grade C Periodontitis in Young Individuals

Author, Year	Study design, Follow-up	Study population, (male/female)	intervention	Clinical/ Microbiological parameters	Host Response (immunity)
Annaji.S et.al. 2016 22	Spilt-mouth 3 months	N = 15 (6/9) LAgP and GAgP at least one tooth with probing pocket depth ≥ 5 mm in each quadrant	(1) SRP alone (2) SRP + Diode Laser (810 nm) (3) SRP + PDT on “0” day (4) SRP+ PDT on “0”, 7^th^ and 21^st^ day	PI BI PD CAL A.a, BPB	
Talmac AC et.al. 2019 21	split-mouth 3 months	N = 26 (14/12) GAgP (Stage III and IV, Generalized, Grade C) ≥ 5 mm of attachment loss around at least seven teeth, excluding first molars and central incisors.	(1) Only SRP group (SRP-control). (2) SRP + Er,Cr:YSGG laser group (SRP + Er,Cr:YSGG). (3) SRP + diode laser (940 ± 15 nm) group (SRP + diode).	PI GI BOP PD CAL	IL-1β IL-8 TNF-α
Talmac AC et.al. 2022 19	split-mouth 3 months	N = 26 (14/12) GAgP (Stage III and IV, Generalized, Grade C) ≥ 5 mm of attachment loss around at least seven teeth, excluding first molars and central incisors.	(1) Only SRP group (SRP-control). (2) SRP + Er,Cr:YSGG laser group (SRP + Er,Cr:YSGG). (3) SRP + diode laser (940 ± 15 nm) group (SRP + diode).	PI GI BOP PD CAL	IL-1β IL-37
Ertugrul AS et.al. 2017 20	split-mouth 1 month	N = 13 (6/7) GAgP (Armitage 1999)	4 different quadrants (1) Only SRP (Control-Er,Cr:YSGG) (2) SRP+ Er,Cr:YSGG laser (Test-Er,Cr:YSGG) (3) SRP+ 940 ± 15nm diode laser (Test-Diode) (4) Only SRP (Control-Diode)	PD PI GI BOP CAL GCF amounts	Human β defensin-1, IL-1β
Matarese G et.al. 2017 24	split-mouth, RCT 15d, 30d 60d, 365d	N = 31 (14/12) GAgP (Armitage 1999)	(1) SRP+diode laser (810nm) (2) SRP alone	PD BOP CAL FMPS (%) Orange/red complex	IL-1β IL-10 TNF-α
Anwar SK et.al. 2024 23	RCT 1 month 3 months	N = 50 (22/28) stage III grade C CAL ≥ 5, PD ≥ 6 mm and rapid rate of bone loss showed by panoramic radiograph	(1) SRP+diode laser (980 nm) (2) SRP+ systemic antibiotic administration (SPM+MTZ)	PD CAL P.g, A.a	
Doğan ^B et.al. 2022 18	RCT 10 days 6 weeks 3 months 6 months	N = 18 (10/8) S3GCP PD ≥6mm and CAL ≥5mm in at least three teeth in both quadrants of the maxilla	(1) MWF alone (2) MWF +DL(810 ±5nm)	PD PI GI CAL BOP P.g, C.r, T.d A.a, P.i, T.f	TE TC
TAN Yani, CUI Xu 2022 28	3 months	N = 97 (58/39) GAgP	(1) MWF (2) MWF+ Nd: YAG laser	BI, GI, BOP PD, TM, PI AL	RANKL OPG
SHI Xuexue 2021 32	RCT 3 months 6 months	N = 60 (25/35) GagP and LAgP	(1) SRP (2) SRP+ Er: YAG laser	BI, PD, MD	
Chawla K et.al. 2022 37	RCT 3 months 6 months 9 months	N = 20 (7/13) CAL ≥ 5mm around at least seven teeth, excluding first molars and central incisors	(1) SRP+APDT (2) SRP+ Er,Cr:YSGG laser (3) SRP alone	PD CAL GI	
ZHENG Ying, LIU Xue, ZHANG Hao 2019 38	split-mouth 1 month 3 months	N=20 (7/13) GAgP	(1) SRP (2) SRP+ Er: YAG+ Nd: YAG	PD BI	
YANG Ting, ZHANG Pengfei, XU Yin et al. 2019 39	RCT 6 weeks 12 weeks	N=78 (40/38) AgP	(1) SRP (2) SRP+ Er: YAG (3) SRP+ Er: YAG+ Nd: YAG	VAS PD BI AL	
Rodrigues RD et.al. 2023 45	double-blinded split-mouth RCT 3 months	N=14 (6/8) Stage III Generalized, Grade C	(1) SRP+ APDT (twice times) (2) SRP+ Simulated APDT	PD CAL GR	
Moreira A L et.al. 2015 51	split-mouth RCT 1month 3 months	N=20 GAgP	(1) SRP+ APDT (four times) (2) SRP	PD, CAL GR, BOP 40 subgingival species	IL-1β IL-10 TNF-α
Costa Coelho TDR et.al. 2023 50	split-mouth RCT 90 d	N=11 (7/13) Stage III Grade C	(1) SRP+ APDT (twice times) (2) SRP+ Simulated APDT	PD, CAL BOP, FI CEJ-GM	
Borekci T et.al. 2019 46	Singled-centered RCT 63 days	N=24 GAgP	(1) NTP (2) NPT+ APDT (twice times)	PI, TM, SBI PD, RAL, GR P.g, P.i T.f, T.d	
Andere NMRB et.al. 2022 47	RCT 3 months 6 months 12 months	N=46 (6/40) Stage III and IV, Grade C	(1) NTP+APDT (four times) (2) OPD+SRP	GR, GI, BOP PD, CAL, PI P.g, A.a VAS	IL-1β、IL-10 IL-4 TNF-α IFN-γ
Bechara AN et.al. 2018 48	RCT 3 months 6 months	N=32 GAgP	(1) UD+UPD (2) UD +UPD+CLM (3) UD+UPD+APDT (four times) (4) UD+UPD+CLM+APDT (one times)	PD, CAL GR, BOP FMBS (%) FMPS (%)	
Al-Khureif AA et.al. 2022 49	RCT 3 months 6 months	N = 18 Stage III and IV, Grade C	(1) UD+ APDT (four times) (2) UD+MTZ+AMX	PI, PD CAL BOP%	IL-10 IL-17
